# Use of fuzzy edge single-photon emission computed tomography analysis in definite Alzheimer's disease - a retrospective study

**DOI:** 10.1186/1471-2342-10-20

**Published:** 2010-09-01

**Authors:** Robert Rusina, Jaromír Kukal, Tomáš Bělíček, Marie Buncová, Radoslav Matěj

**Affiliations:** 1 Department of Neurology, Thomayer Teaching Hospital and Institute for Postgraduate Education in Medicine, Prague, Czech Republic; 2 Department of Software Engineering in Economy, Faculty of Nuclear Science and Physical Engineering, Czech Technical University, Prague, Czech Republic; 3 Department of Nuclear Medicine, Institute for Clinical and Experimental Medicine, Prague, Czech Republic; 4 Department of Pathology and Molecular Medicine, Thomayer Teaching Hospital, Prague, Czech Republic

## Abstract

**Background:**

Definite Alzheimer's disease (AD) requires neuropathological confirmation. Single-photon emission computed tomography (SPECT) may enhance diagnostic accuracy, but due to restricted sensitivity and specificity, the role of SPECT is largely limited with regard to this purpose.

**Methods:**

We propose a new method of SPECT data analysis. The method is based on a combination of parietal lobe selection (as regions-of-interest (ROI)), 3D fuzzy edge detection, and 3D watershed transformation. We applied the algorithm to three-dimensional SPECT images of human brains and compared the number of watershed regions inside the ROI between AD patients and controls. The Student's two-sample t-test was used for testing domain number equity in both groups.

**Results:**

AD patients had a significantly reduced number of watershed regions compared to controls (*p *< 0.01). A sensitivity of 94.1% and specificity of 80% was obtained with a threshold value of 57.11 for the watershed domain number. The narrowing of the SPECT analysis to parietal regions leads to a substantial increase in both sensitivity and specificity.

**Conclusions:**

Our non-invasive, relatively low-cost, and easy method can contribute to a more precise diagnosis of AD.

## Background

Alzheimer's disease (AD) is the most common neurodegenerative dementia. Diagnostic criteria are based mainly on clinically altered cognition. Early diagnosis of AD is crucial for maximizing treatment benefits. Neuroimaging may be helpful in increasing diagnostic precision, but correlations between localized atrophy, mainly in the temporal regions, on MRIs, and AD pathology are still controversial, and promising new techniques like PET amyloid imaging are not in routine use. Low beta-amyloid and elevated tau protein levels in cerebrospinal fluid have been correlated with AD at a sensitivity of 85-94% and a specificity of 83-100% [1]. However, other studies have not been able to confirm these results and widespread consensus is lacking regarding its utility in everyday practice [2].

Single photon emission computerized tomography (SPECT) is a widely used diagnostic method based on analysis of regional cerebral blood flow (rCBF); with restricted rCBF considered to reflect hypometabolism and consequently hypofunction. Typical SPECT AD patterns show reduced rCBF in both temporal and parietal regions, and, in a recent review, were capable of distinguishing AD from healthy controls (sensitivity = 65% - 71%; specificity = 79%) [3]. SPECT studies with autopsy-confirmed diagnoses reported sensitivities of 86 to 95% and specificities of 42 to 73% [4,5]. Since "raw" data needs further treatment, final results from SPECT investigations are, at least partly, operator-dependent and both specificity and sensitivity vary among centers. Because of its low sensitivity and specificity, routine use of SPECT is not recommended for diagnostic purposes [6].

Currently, new methods for signal processing and supervised learning have demonstrated the potential of computer aided diagnostic systems [7,8].

Computer based analysis of SPECT data is of increasing interest in the field. The superiority of 3-dimensional stereotactic surface projection analysis (3D-SSP) over visual inspection for differentiating patients with very early AD from control subjects using brain perfusion SPECT has been reported [9]. The authors found that 3D-SSP had an accuracy of 86.2% for differentiating patients with AD from control subjects when analyzing the posterior cingulate gyri and precunei. In contrast, visual inspection only had an accuracy of about 74.0%. Voxel-based analysis (using specific voxel-based Z score maps) may be helpful in differentiating AD from vascular dementia and non-demented patients using a method which is not influenced by inter-observer differences among radiologists [10]. These procedures, however, necessitate special software applications and are not routinely used in many countries.

Nevertheless, reasonable financial costs and the possibility of using SPECT repetitively for monitoring disease progression, offer arguments for routine use of SPECT, assuming that specificity and sensitivity can be increased through improved data processing.

The aim of our study was to develop a procedure with at least comparable accuracy to the results of visual inspection in differentiating AD patients from controls and at the same time avoid the need for special additional equipment.

## Methods

Our study is based on a post hoc (retrospective) analysis of raw SPECT data, acquired between 2003 and 2005. The data were analyzed with respect for patient privacy and the protocol was approved by the local Ethics Committee.

We enrolled SPECT data from 17 adult patients with definite Alzheimer's disease confirmed by autopsy, as defined by NIA-Reagan Institute criteria as well as the Consortium to Establish a Registry for Alzheimer's disease criteria.

We routinely perform SPECT in patients with cognitive impairment as a routine diagnostic procedure. Therefore, in our setting, the data from SPECT scans and the confirmation of clinical diagnosis of AD were very timely (within a few weeks). All patients included in the study were diagnosed with mild to moderate AD (later confirmed by autopsy) according to NINCDS-ADRDA and DSM-IV criteria; additionally all patients were diagnosed with dementia.

Control cases included 10 patients with amyotrophic lateral sclerosis (ALS), without signs or complaints of cognitive dysfunction, who underwent SPECT and a detailed cognitive evaluation as part of a previously published research protocol [11].

SPECT studies were performed using a standardized protocol, which started 40 minutes after injection with 740 MBq 99mTcHMPAO (hexamethylpropyleneamine-oxime labeled with 99mTechnetium) and used a dual-head gamma camera (DST-XL SOPHA with LEHR collimator). We used filtered back projection (FBP) for image reconstruction. No correction for attenuation was made.

We prepared 3D SPECT brain scans in six consecutive operations: (i) image smoothing, (ii) normalization, (iii) background elimination, (iv) fuzzy edge detection, (v) watershed segmentation, and (vi) region counting.

Image smoothing (i), used a traditional method of noise suppression and was performed with a Gaussian 3D filter with radius as the first parameter.

The second step was oriented toward image intensity normalization (ii) in an interval (0, 1), where unit intensity corresponded to maximum brain activity.

Background elimination (iii) was the next image-processing step. The normalized intensity was compared with a threshold value as the second parameter of data processing. Positive differences were passed while negative ones were set to zero.

The fourth step was fuzzy edge detection (iv) based on Lukasiewicz BL-algebra [12]. Every voxel of the previous 3D image (after step iii) has 6 neighboring voxels; the fuzzy edge intensity (for a given voxel) was defined as the aggregate fuzzy non-equivalence between the voxel and its neighbors. The fuzzy equivalence of two voxel intensities was realized as a bi-residuum in Lukasiewicz BL-algebra. Bi-residuum reaches its unit maximum when the intensities are equal and the value falls to zero when they are opposite. The fuzzy non-equivalence is only a fuzzy negation of equivalence as a complement to the unit value. The fuzzy aggregation of six pairwise non-equivalences was performed via a fuzzy "OR" operator as the maximum function. Applying this procedure to every voxel and its neighbors, we obtain a 3D image of fuzzy edges (iv), which depicted structures with maximum morphological gradients of brain activity.

**Figure 1:**
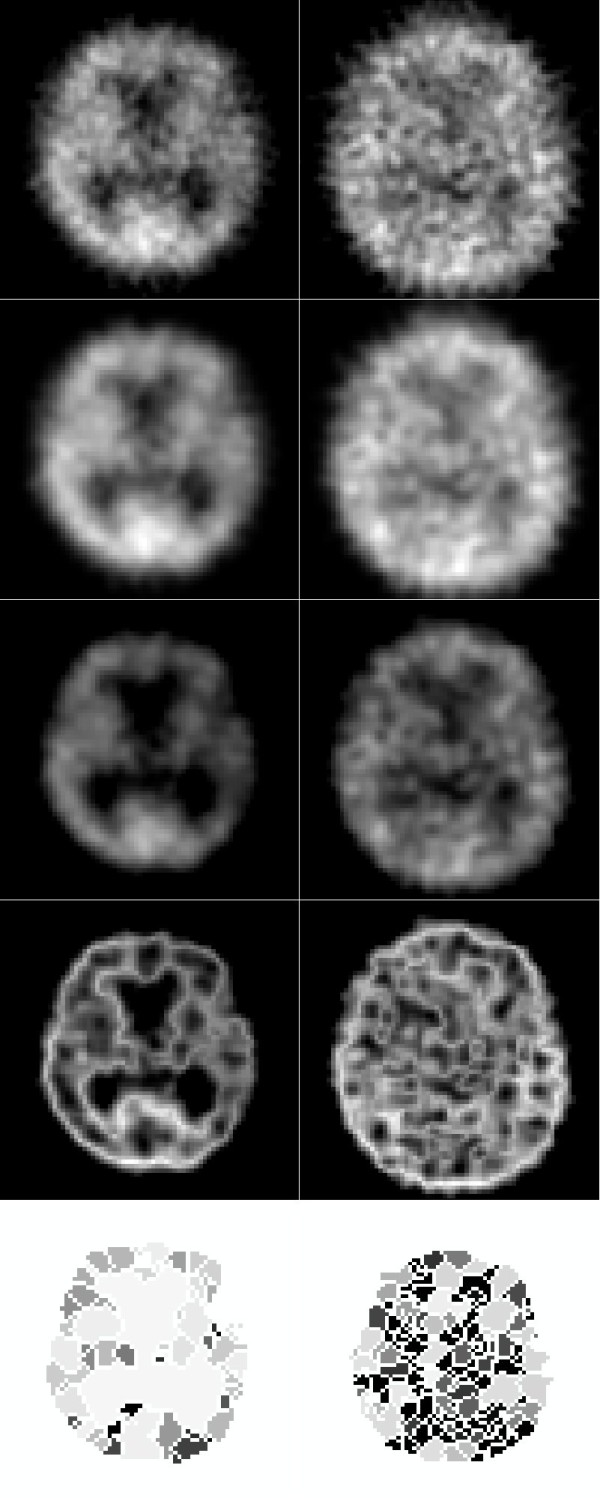
**Processing steps for typical AD patients (left) and controls (right): original SPECT (1st - above), smoothing and normalization (2nd), thresholding (3rd), fuzzy edge detection (4th), watershed (5th - bottom)**.

**Figure 2:**
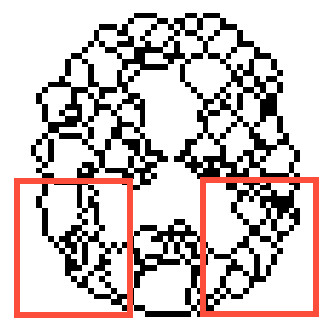
**Final 3D watershed in a typical AD patient with 40 spatial regions (central slice)**.

**Figure 3:**
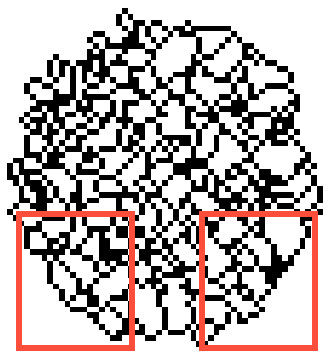
**Final 3D watershed in a typical control with 70 spatial regions (central slice)**.

Edge contours with high intensity can help in image decomposition based on brain activity. The process of segmentation was automated using a standard 3D watershed transform (v) and constituted step five. The watershed method [13] is a tool for the digital image segmentation, which is based on the study of local minima and their basins of attraction. Watershed shapes in 3D consists of points where two basins of attraction are at least in their neighborhood. Sub-results of this procedure are demonstrated in Figure 1 for a central slice of the whole SPECT image of typical AD and control brains. The resulting 3D image of a parietal ROI was labeled to demarcate the regions and watershed borderlines (Figures 2, 3).

The last image processing step (vi) counts the total number of separated 3D regions into regions-of-interest (ROIs) centered in the left and right parietal lobes.

All statistical calculations were performed using Matlab Statistical Toolbox from MathWorks Inc. Statistical characteristics were evaluated as point estimates together with 95% confidence intervals. The null hypothesis of the mean value equity was tested using the two-sample Student's t-test. Due to the relatively small groups of AD patients and controls, the 'leave-one-out' method [14] of cross-validation was used to obtain mean values of processing parameters and their standard deviations. Finally, the null hypothesis of the mean value equity was tested using the two-sample Student's t-test and the sensitivity and specificity of proposed method were estimated.

## Results

The study included 17 adult patients, where definite Alzheimer's disease was confirmed by autopsy using the NIA-Reagan Institute criteria (neocortical tangles score Braak V-VI), as well as the Consortium to Establish a Registry for Alzheimer's Disease criteria (CERAD plaque score frequent), and confirmed using specific monoclonal antibodies against hyperphosphorylated tau protein and amyloid beta peptide. Basic patient characteristics are summarized in Tables 1, 2.

**Table 1:** Demographic data of patients and controls

Patient	Gender	Age (years)	MMSE	Braak stage	Comorbidity
1	M	78	23	VI	arterial hypertension
2	F	78	17	VI	alcohol abuse 10 years earlier
3	F	57	25	V	asthma, glaucoma
4	F	79	20	VI	arterial hypertension, coronary by-pass
5	M	74	19	VI	NA
6	M	76	20	VI	hypertension, minor stroke, diabetes, atrial fibrillation
7	F	83	10	VI	NA
8	F	83	20	VI	arterial hypertension
9	M	68	20	VI	NA
10	M	84	18	VI	NA
11	M	83	16	V	arterial hypertension, diabetes, myocardial infarction, hyperlipidemia
12	M	80	18	VI	arterial hypertension, subarachnoidal hemorrhage 30 years earlier
13	M	81	20	VI	NA
14	M	80	21	V	prostatic hypertrophy
15	F	86	16	VI	ischemic heart disease, diabetes
16	F	80	15	V	hypertension, atrial fibrillation
17	F	87	20	V	hypertension

**Table 2:** Basic statistical characteristics of patients and controls

Group	Characteristics	Mean	SD	Minimum	Maximum
AD	F/M	8/9			
	Age (years)	78.5	7.2	56	87
	Duration onset-diagnosis (months)	43	19	12	85
	Duration diagnosis-death (months)	19	13	1	50
	MMSE	18.7	3.4	10	25
Control	F/M	3/7			
	Age (years)	55.9	7.6	39	70
	MMSE	30	0	30	30

AD: Alzheimer's disease

MMSE: Mini Mental State Examination

F: female M: male subjects

NA: not applicable

Duration onset-diagnosis: time span from manifestation of first AD signs to clinical diagnosis confirmation

Duration diagnosis-death: time span from clinical diagnosis of AD to death

The 3D SPECT scans of 17 AD patients and 10 controls were analyzed as 3D matrices of 128×128×128 voxels using the described method with left and right parietal lobe ROIs having individual sizes of 22×29×3 voxels. Basic characteristics such as gender (F/M), age, Onset-Diagnosis, Diagnosis-Death, MMSE and the Braak stage are summarized in Tables 1, 2. The cross-validation using the 'leave-one-out' method had two main results.

The mean values of processing parameters and their standard deviations were estimated. The optimum radius of Gaussian filtering was 0.8997 ± 0.0637, the threshold value was 0.2761 ± 0.0511 and the critical number of regions was 57.11 ± 0.32. There were 15 true positive cases and 8 true negative cases after cross-validation. Adequate sensitivity was 88.2% and the specificity reached 80.0%.

**Figure 4:**
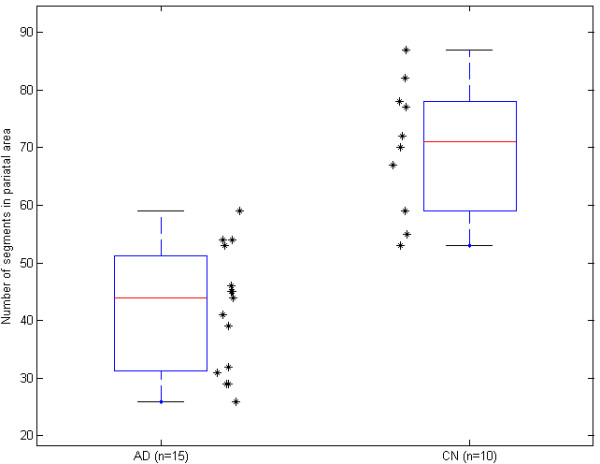
**Box-plot of watershed region number in AD and controls (CN)**.

The mean values of parameters from cross-validation were used for the final statistical testing using the two-sided two-sample t-test. Results of posterior statistical analysis are presented in Table 3 and Figure 4 as the number of watershed regions in the ROIs.

**Table 3:** Number of watershed regions for AD and controls

Characteristics	AD patients Value [95% CI]	Controls Value [95% CI]
Mean	41.4 [33.7, 47.9]	68.1 [59.4, 76.8]
Std. deviation	9.9 [6.7, 17.9]	12.1 [8.3, 22.1]
Sensitivity (%)	94.1 [59.6, 98.3]	NA
Specificity (%)	NA	80.0 [44.4, 97.5]

NA: not applicable

CI: confidence interval

AD: Alzheimer's disease

SPECT data were recorded by one investigator (M.B.) while statistical analysis was performed separately two others (T.B., J.K.) anonymously without any information about the patients.

The testing criterion value was *t *= 6.187 and the probability value was *p *= 3.8×10^-7 ^(i.e. p < 0.01), which means significantly fewer numbers of watershed regions in the AD group compared to controls. The optimum threshold for watershed regions was set to 57; the number of true positive cases was *TP *= 16 and the number of true negative cases was *TN *= 8. Thus, the method based on fuzzy edge detection and watershed transform reached a sensitivity of 94.1% and a specificity of 80.0% for AD patients and controls.

## Discussion

AD diagnosis is based mainly on cognitive evaluation and a definite diagnosis requires neuropathological findings of beta amyloid deposits and neurofibrillary tangles.

AD is essentially a disease of the elderly and most patients have co-morbidities that may affect cognition. It is therefore important to emphasize that all our patients had neuropathologically proven "pure" Alzheimer's disease. In our retrospective study, we excluded all patients with vascular encephalopathy and other neurodegenerative brain disease. We also considered several co-morbidities in the patients selected to our study (listed in Table 1) but these were assessed as non-relevant in terms of their influence on the patient's cognitive performance.

Selection of controls involved two principal conditions: first, control cases had to have normal cognitive status and second, SPECT data needed to be available. Given the fact that SPECT utilizes a radioactive marker, it would be difficult to propose such an examination for healthy volunteers. We used post hoc data from an established register, which required taking into account that our controls might be younger than the AD patients (Table 1, 2) and could constitute a limitation of the study.

Finally, we decided to use data from 10 patients with amyotrophic lateral sclerosis (ALS) as controls. The patients were without noticeable co-morbidities, and had been assessed as part of a previously published study [11]. At the time of SPECT scanning, the cognitive status of the controls was normal and patients had no subjective memory and/or cognitive complaints. We considered them as cognitively normal subjects. Five control patients died before the end of the study and selected autopsies showed no significant AD related pathology.

In order to avoid possible bias, SPECT data acquisition and diagnostic evaluation were strictly separated from SPECT data analysis. The investigators (TB, JK) involved in SPECT data treatment were blinded and did not have access to any clinical and/or imaging details about the patients.

We propose a new method of SPECT image processing that could enhance the accuracy of an AD diagnosis. We developed a new approach for treating 'raw' SPECT data. The combination of digital filtering, fuzzy edge detection and watershed method facilitates detection of hypo-perfusion in a smaller number of localized segments.

Respecting the typical temporo-parietal SPECT pattern of AD, we hypothesized that critical differences between AD patients and controls could be found in the parietal regions. Focusing SPECT analysis on the parietal regions substantially increases both sensitivity and specificity, and approaches the 80% levels recommended by the Reagan Biomarker Working Group [15].

In SPECT of AD patients, perfusion in the posterior cingulate is also significantly decreased. However, it is difficult to distinguish a slight decrease in rCBF during early AD by visual inspection [16]. Moreover, according to a longitudinal SPECT study [17], decreases in rCBF adjusted for relative flow distribution, by normalization of global cerebral blood flow in the posterior cingulate gyrus and precuneus, became ambiguous as the disease progressed.

As demonstrated in Figure 1, the spatial resolution of our procedure is limited, therefore, and respecting the cited arguments, we decided to analyze 'traditionally used' parietal regions. This decision was supported by a recent study [18] that described a significant correlation between tau or phospho-tau concentrations in cerebrospinal fluid and perfusion in the left parietal cortex in AD patients.

In our study, the total number of regions was the only criterion for patient classification. The novelty and efficiency of our method is based on a combination of a fuzzy edge detector, watershed transform, and orientation toward activity separation of parietal lobe domains; other operations were necessary to reduce sensitivity to noise and artifacts.

## Conclusions

SPECT data can be easily manipulated using available software; underscoring that extra software and/or manual corrections of raw SPECT data is not required; therefore, our method can be easily used by clinicians. Additionally, it offers earlier and more precise AD diagnoses with the associated patient benefits, and it can be done without significantly increased costs.

## Abbreviations

The list of abbreviations used is as follows: SPECT: single photon emission computerized tomography; AD: Alzheimer's disease; ROI: regions-of-interest; rCBF: regional cerebral blood flow; CERAD: Consortium to Establish a Registry for Alzheimer's disease; ALS: amyotrophic lateral sclerosis

## Competing interests

The authors declare that they have no competing interests.

## Authors' contributions

RR and JK made substantial conceptual contributions to the design of the study, analysis and interpretation of data, and contributed to drafting of the manuscript. TB was involved in data analysis and interpretation, and developed mathematical tools for SPECT data analysis. MB was involved in acquisition of SPECT data, and RM performed neuropathological verifications and gave critical revision of the manuscript regarding important intellectual content. All authors have read and approved the final version of the manuscript.

## Pre-publication history

The pre-publication history for this paper can be accessed here:

http://www.biomedcentral.com/1471-2342/10/20/prepub
